# Preparation and Anti-Icing Properties of Zirconia Superhydrophobic Coating

**DOI:** 10.3390/molecules29081837

**Published:** 2024-04-18

**Authors:** Jiahui Zhou, Haikun Zheng, Wei Sheng, Xiaoru Hao, Xinmin Zhang

**Affiliations:** 1 Hami Vocational and Technical College, Hami 839001, China; woshizjhzhoujiahui@163.com; 2 School of Mechanical and Power Engineering, Henan Polytechnic University, Jiaozuo 454003, China; weisheng@163.com (W.S.); xiaoru408@126.com (X.H.); zhangxm@hpu.edu.cn (X.Z.)

**Keywords:** zirconium oxide, docosanoic acid, superhydrophobicity, self-cleaning, anti-icing

## Abstract

Zirconia (ZrO_2_) is a ceramic material with high-temperature resistance and good insulating properties. Herein, for the first time, the surface of ZrO_2_ was modified with docosanoic acid (DCA) to improve its self-cleaning and hydrophobic properties. This surface modification transformed the surface of ZrO_2_ from hydrophilic to superhydrophobic. A two-step spraying method was used to prepare the superhydrophobic surface of ZrO_2_ by sequentially applying a primer and a topcoat. The primer was a solution configured using an epoxy resin as the adhesive and polyamide as the curing agent, while the topcoat was a modified ZrO_2_ solution. The superhydrophobic surface of ZrO_2_ exhibited a contact angle of 154° and a sliding angle of 4°. Scanning electron microscopy, X-ray diffraction, energy-dispersive X-ray spectroscopy, thermogravimetric analysis, and other analytical techniques were used to characterize the prepared zirconia particles and their surfaces. Moreover, results from surface self-cleaning and droplet freezing tests showed that DCA-modified ZrO_2_ can be well combined, and its coatings show good self-cleaning and anti-icing properties on TA2 bases.

## 1. Introduction

Zirconia (ZrO_2_) has stable chemical properties, a high melting point, a high electrical resistivity, a high refractive index, and a low coefficient of thermal expansion. It has become an important additive for high-temperature resistant materials, ceramic insulation materials, and coatings [1,2,3]. Zirconia exhibits an excellent performance and using it to prepare superhydrophobic coatings can further enhance performance, such as frost suppression [4,5,6], anti-icing [7], and self-cleaning [8,9], and can be applied in aerospace [10,11], medical health [12], solar power generation [13], and other fields. The common methods for preparing super hydrophobic coatings include electrodeposition [14,15,16], laser processing [17,18], immersion [19,20], sol–gel [21], spraying [22,23], etc. The preparation of superhydrophobic surfaces using spraying techniques requires the formation of micro and nano structures on the surface using micro and nanoparticles, in order to achieve the lotus leaf effect of superhydrophobicity. The preparation of micro and nano particles in superhydrophobic coatings via spray [24] coating requires modification with low surface energy substances to prepare superhydrophobic materials. The micro and nanoparticles in the superhydrophobic coatings prepared using the spraying method need to be modified with low surface energy substances to prepare superhydrophobic materials, among which common micro and nanoparticles are graphene oxide [25], carbon nanotubes [26], silicon oxide [27], titanium oxide [28], and zirconia [29].

There are many experts and scholars who have modified zirconia to prepare superhydrophobic materials. Ubong Eduok et al.’s [30] superhydrophobic cotton fabrics were prepared via an immersion method. First, zirconia nanoparticles were modified using hydroxyethyl cellulose as a modifying material. Second, the modified Zr(OC_2_H_5_)_4_ particles were mixed with polydimethylsiloxane to obtain a mixed solution. Finally, the cotton fabric was immersed in the mixed solution to obtain superhydrophobic cotton fabric. The contact angle in the prepared composite-coated fabric was 154°. Ying Shen et al.’s [16] polyvinylidene fluoride (PVDF)-modified ZrO_2_ was used to prepare superhydrophobic PVDF-PbO_2_-ZrO_2_ electrodes with a stable performance and a high efficiency via an electrodeposition method. The coated products were prepared with a maximum contact angle of 156°, which is good for wastewater removal. Uzma K.H. Bangi et al. [31] use the method of co-precursor sol–gel, using ZrPro as the precursor and trimethylchlorosilane, hexamethyldisilane, and perfluorodecyltriethoxysilane as the three silane coupling agents. Prasanth and Vuppalapati Giri et al. [32] prepared PVDF-modified ZrO_2_ nanoparticle superhydrophobic coatings using a spray coating method. Firstly, the modified nanoparticles were dispersed into an acetone solution. Secondly, the solution was sprayed onto a glass slide and was dried to obtain a superhydrophobic coating with a maximum contact angle of 153°. Jialin Yang [33] prepared a coating with polydimethylsiloxane (PDMS), which exhibited good salt spray resistance for up to 210 h and a contact angle of 109.3°. Yangxi Zhang [34] used a photolithographic three-dimensional (3D) microprinting method to prepare polytetrafluoroethylene surfaces with micro/nanostructures. Haiqiao Zhang [35] successfully replicated a superhydrophobic surface with a micro/nano structure of lotus leaves on polyurethane acrylate using UV nanoimprinting technology. There are two main types of changes in the hydrophobicity of zirconia. The first type is the laser processing of the zirconia surface to make the surface have micro and nano structures, similar to the lotus leaf state, to achieve hydrophobicity directly. The second type is to modify micro and nano particles with low surface energy substances, such as changing the hydrophobicity of zirconia, and using spraying, sol–gel, immersion, and other methods to make superhydrophobic surfaces.

In the preparation process of superhydrophobic coatings, low surface energy substances are commonly used to modify nanomaterials, including fatty acids, silane coupling agents, and fluorosilanes. Stearic acid [36], palmitic acid [37], myristic acid [38], and so forth are commonly used fatty acids [39,40]. Methyltrimethoxysilane [41], 1H,1H,2H,2H-perfluorodecyltriethoxysilane [42], trimethoxysilane [43], and so forth are commonly used silane coupling agents.

In summary, research on zirconia superhydrophobic surfaces can mainly be divided into two aspects. Firstly, the commonly used low surface energy substances for preparing zirconia superhydrophobic surfaces, include silane coupling agents and fluorosilanes, with contact angles around 155°. However, research on fatty acid-modified zirconia is still insufficient. Secondly, research on the superhydrophobic surface of zirconia mainly focuses on the performance of wastewater treatment and salt spray prevention and there is a lack of research on anti-icing. For the first time, docosanoic acid [44] (C_22_H_44_O_2_; DCA), a fatty acid, was used as a low surface energy substance to modify zirconia micro and nanoparticles for the sake of environmental protection. The superhydrophobic surface was prepared using a two-step spraying method and was sprayed onto the surface of TA2 pure titanium metal square base.

## 2. Results and Discussion

### 2.1. Surface Preparation and Characterization

**Figure 1:**
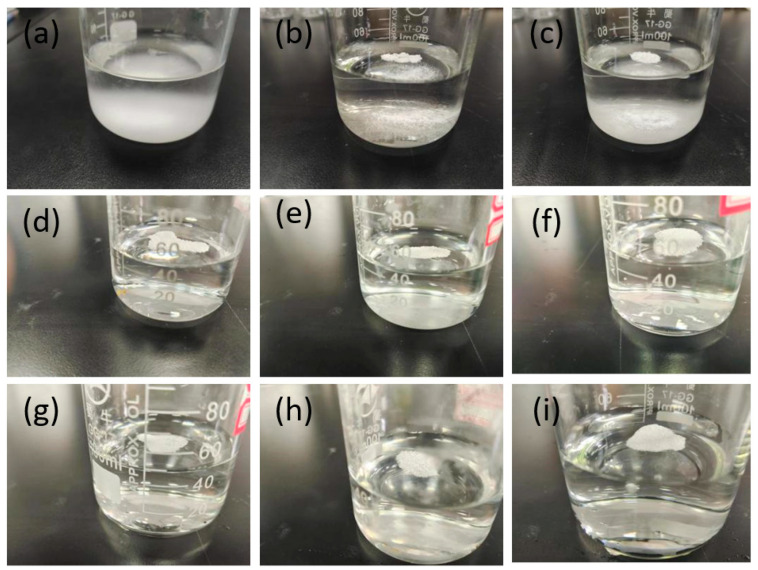
Suspension of different ratios of materials in water, between (**a**–**i**) are nanoparticles from DCA0-ZrO_2_ to DCA8-ZrO_2_.

Before preparing the superhydrophobic surface, immersion tests of materials with different modification ratios were performed. Results showed that when zirconia without DCA modification, DCA01-ZrO_2_, and DCA02-ZrO_2_ are placed in water, precipitation occurs. The particles of the precipitated materials are unmodified or incompletely modified materials, as shown in Figure 1a–c. However, when DCAx-ZrO_2_, x = 3–8, is placed in water, no precipitation occurs, as shown in Figure 1d–i. Therefore, it can be concluded that when 0.3 g or more of DCA is added to 6 g of zirconia particles and the resulting material is placed in water for 7 days, no obvious precipitation occurs. Therefore, zirconia is hydrophobic only if there is a suitable ratio. In the surface-coating process, hydrophobic nanoparticles that do not precipitate in water are sprayed onto TA2 to prepare superhydrophobic surfaces. Therefore, in the next experiments, the main study was of DCA3-ZrO_2_ to DCA8-ZrO_2_-modified zirconia particles to prepare coated surfaces.

The contact angle of DCA0-ZrO_2_ in one stage of the contact angle test is about 38.6° and the contact angle measurement process found that the water droplets just fall down on the surface when the droplets show a spherical shape and when the water droplets spread on the surface after 3–5 S; when the time passes after 60 S, the surface shows a completely wetting state and the droplets are completely spread on the surface. The contact angles of DCA1-ZrO_2_ and DCA2-ZrO_2_ superhydrophobic surfaces are 150.7° and 151.7°, respectively, and, although their contact angles are greater than 150°, the sliding angle is greater than 10° and fluctuates a lot. Therefore, DCA0-ZrO_2_ to DCA2-ZrO_2_ do not meet the definition of superhydrophobicity.

**Figure 2:**
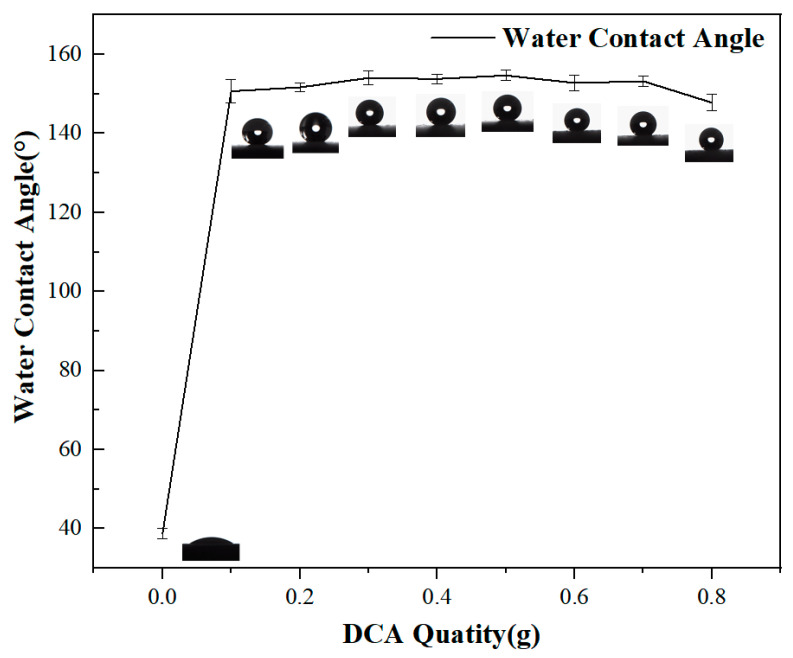
Water contact angle.

**Figure 3:**
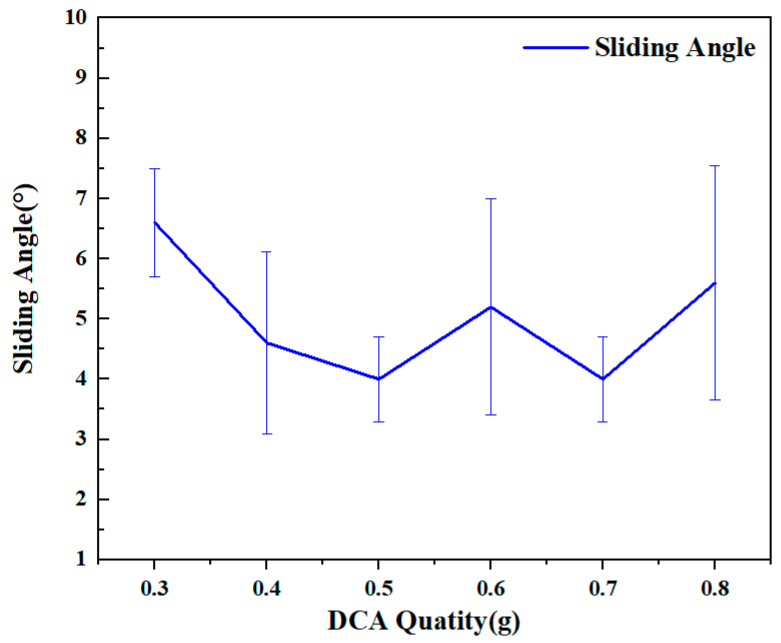
Sliding angle.

During the wettability testing process, it was found that superhydrophobicity could be achieved between DCA3-ZrO_2_ and DCA7-ZrO_2_. The contact angle remained stable at 154° in 0.3–0.5 g and the sliding angle gradually decreased from 6.6° in the DCA3-ZrO_2_ coating to 4° in the DCA5-ZrO_2_ coating. The contact angle showed a decreasing trend from DCA5-ZrO_2_ to DCA7-ZrO_2_, with a sliding angle of 4° and a contact angle of 153.2° in the DCA7-ZrO_2_ coating. For DCA8-ZrO_2_, the contact angle of the coating surface decreased to 148°, the sliding angle increased to 5.6°, and the hydrophobicity of the surface decreased significantly, as shown in Figure 2 and Figure 3. In summary, it was determined in the study that the ratio of 0.5 g DCA and 6 g ZrO_2_, i.e., DCA5-ZrO_2_, had better hydrophobicity. Therefore, SEM, FTIR, EDS, XRD, and TGA analyses were conducted on the surface and particles in their respective proportions.

**Figure 4:**
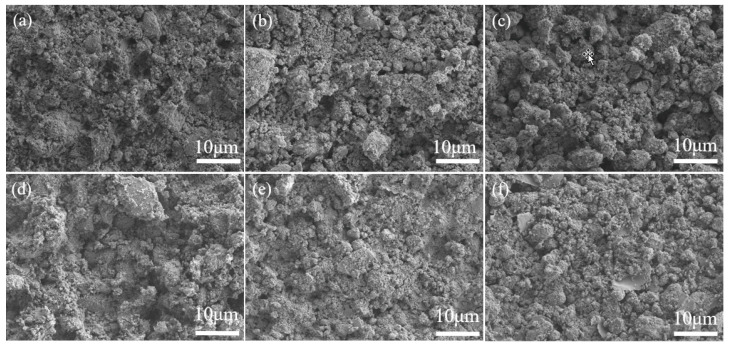
SEM images with different ratios of modified materials. (**a**–**f**) are coatings of modified materials from DCA3-ZrO_2_ to DCA8-ZrO_2_, respectively.

The SEM images of the coating of DCA-modified ZrO_2_ are shown in Figure 4. As shown in Figure 4, the surfaces of the DCA-modified nanoparticles analyzed from 0.3 g to 0.8 g have tiny cracks and pores, while the surface particles are relatively uniformly distributed on the metal surface. As the amount of DCA added increases to reach the supersaturated state in solution, the particle aggregation in the solution improves and, simultaneously, excess DCA covers the micro/nanostructure of zirconium, reducing the hydrophobicity of the surface of zirconium.

**Figure 5:**
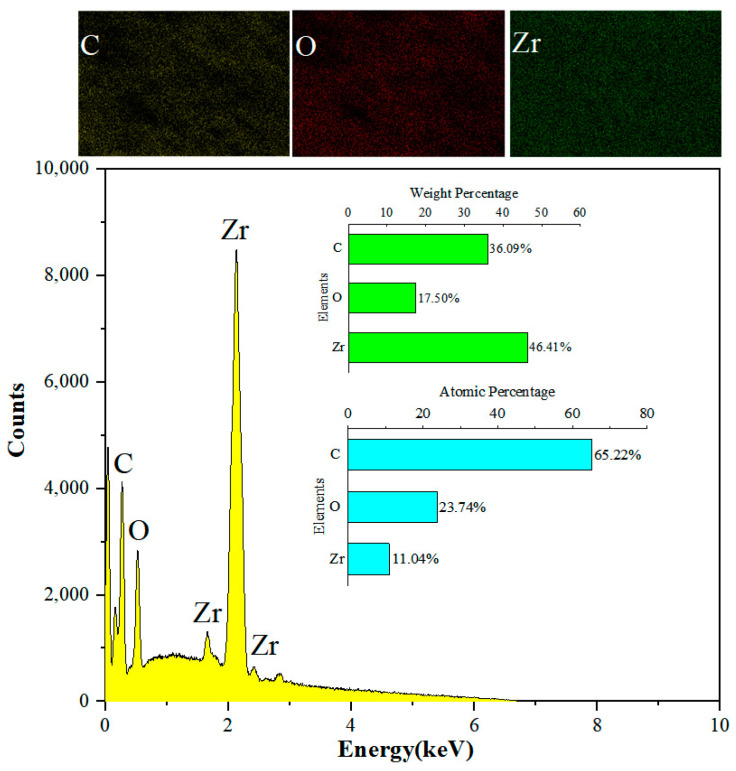
EDS of DCA5-ZrO_2_.

Firstly, 0.5 g of DCA and 6 g of ZrO_2_ particles were put into a beaker and stirred to react fully, to produce a superhydrophobic coating surface. EDS tested the superhydrophobic coatings with DCA5-ZrO_2_, using a surface sweeping method. The test was repeated four times and elemental analysis was performed. The main elements detected were C, O, and Zr; their weight percentages were 36.09%, 17.50%, and 46.41%; and the atomic number ratios were 65.22%, 23.74%, and 11.04%, respectively. As shown in Figure 5, the atomic number of the C element is higher than that of the Zr element, because the relative molecular mass of the C element is smaller than that of the Zr element, reflecting that the modified zirconia contains a large amount of carbon, indicating that DCA has successfully modified zirconia.

**Figure 6:**
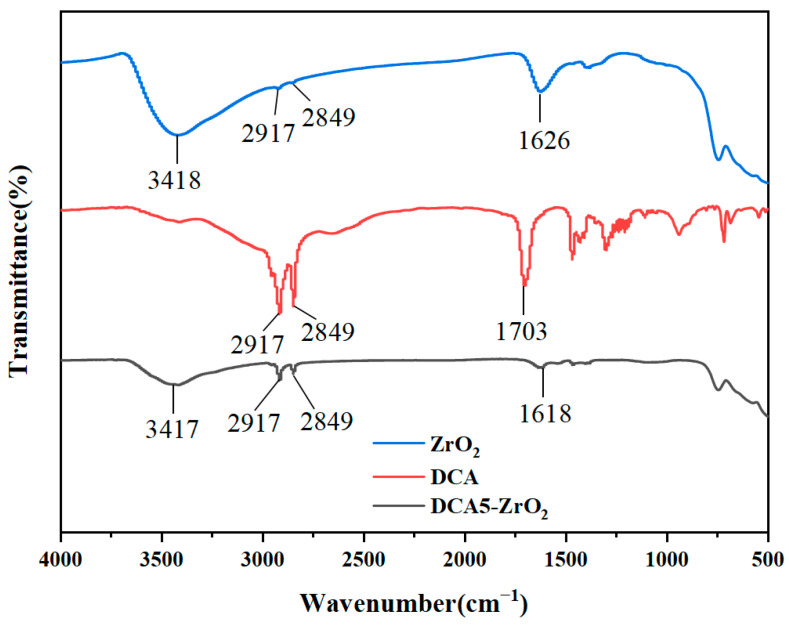
FTIR spectra of ZrO_2_, DCA, and DCA5-ZrO_2_.

The FTIR spectra of ZrO_2_, DCA, and DCA5-ZrO_2_ powders are shown in Figure 6. In the FTIR spectrum of modified and unmodified zirconia nanoparticles, the peak of -OH adsorbed around zirconia is detected at 3417 cm^−1^. The peaks corresponding to the stretching vibration of -CH_2_- and -CH_3_ are observed at 2917 cm^−1^ and 2849 cm^−1^ in the FTIR spectrum of DCA05-ZrO_2_, respectively. However, in the FTIR spectrum of ZrO_2_, the peaks of -CH_2_- and CH_3_ are detected, but are very weak, indicating that DCA has successfully modified ZrO_2_. In the FTIR spectrum of DCA5-ZrO_2_, the peaks of -CH_2_ and -CH_3_ are observed at 2917 cm^−1^ and 2849 cm^−1^, respectively. The peaks of -CH_2_ and-CH_3_ are observed in the FTIR spectrum of ZrO_2_, but with low intensities, indicating that DCA has successfully modified ZrO_2_. FTIR results indicate that ZrO_2_ was successfully modified by DCA.

**Figure 7:**
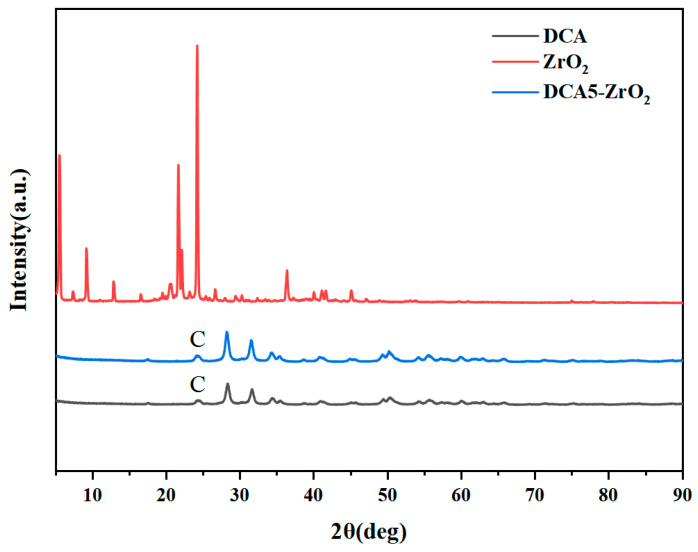
XRD patterns of DCA, ZrO_2_, and DCA5-ZrO_2_.

Figure 7 displays the XRD patterns of both DCA and DCA5-ZrO_2_. In all XRD patterns, a peak corresponding to C is observed at ~25°. DCA5-ZrO_2_ represents a hybrid modified by DCA, and the XRD patterns obtained from ZrO_2_ nanoparticles remain largely unchanged compared to those of pristine ZrO_2_ nanoparticles. This similarity arises because the modified ZrO_2_ nanoparticles were encapsulated by DCA, resulting in detected crystalline structures resembling those of ZrO_2_. The crystal structure is similar to that of ZrO_2_, presenting differences from pristine ZrO_2_.

**Figure 8:**
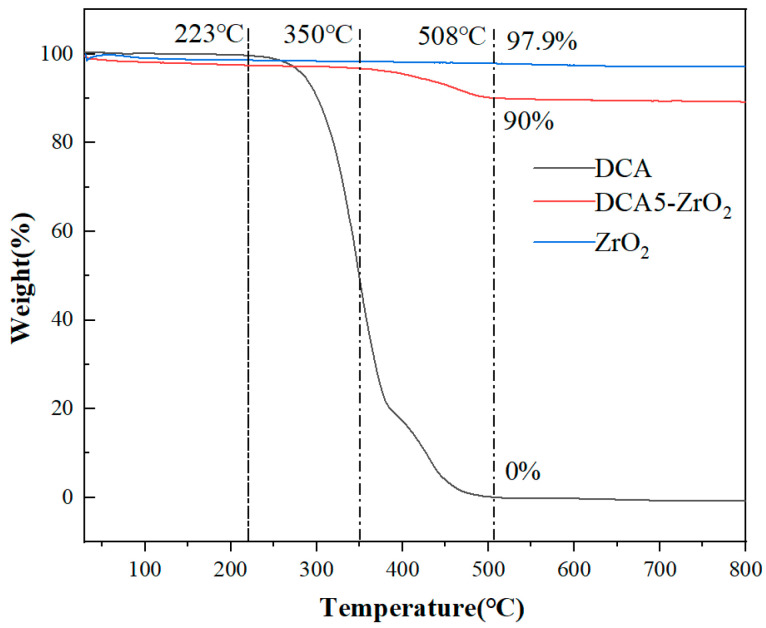
TGA curves of DCA and DCA5-ZrO_2_.

The thermal stability of DCA and DCA5-ZrO_2_ was determined via TGA from 30 °C to 800 °C at a heating rate of 10 °C/min in a nitrogen environment. According to TGA, DCA exhibits a weight loss of ~0.4% at temperatures below 223 °C, due to water evaporation. As the temperature gradually increases, the weight loss of DCA increases and DCA exhibits a weight loss of 51% at 350 °C and 0% at 508 °C, indicating that DCA completely decomposes at 508 °C. At 223 °C, ZrO_2_ experiences a weight loss of 1.4%, due to water evaporation. With prolonged exposure to higher temperatures, the change in the quality of ZrO_2_ remains minimal. In measurements, the accuracy error is within 1% of the observed change. However, at 508 °C, the weight loss due to water evaporation increases significantly to 97.9%. DCA5-ZrO_2_ exhibits a weight loss of 2.6% at 223 °C, due to water evaporation. As the temperature gradually increases to 350 °C, DCA5-ZrO_2_ exhibits a weight loss of 3.3%, followed by an increase in the weight loss rate at 508 °C, which tends to stabilize the weight loss of the cumulative total of 10%, as shown in Figure 8. This indicates that the thermal stability of DCA5-ZrO_2_ and DCA significantly increases. The weight loss disparity between ZrO_2_ and DCA5-ZrO_2_ at 508 °C was 7.9%. This discrepancy suggests a notable enhancement in thermal stability for DCA5-ZrO_2_ compared to ZrO_2_ alone. Consequently, the data highlight a significant improvement in thermal stability conferred by DCA in DCA5-ZrO_2_.

### 2.2. Surface Self-Cleaning Evaluation

**Figure 9:**
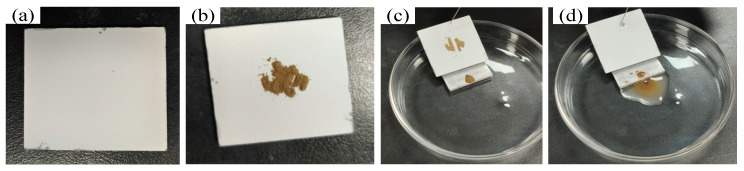
Self-cleaning test of DCA5-ZrO_2_. (**a**) Original surface, (**b**) dusty surface, (**c**) surface during self-cleaning, and (**d**) surface after self-cleaning.

Surface self-cleaning is an important property of superhydrophobicity surfaces. In the self-cleaning test, the surface was prepared with DCA5-ZrO_2_, placed at an angle in a glass container with a 3 mm high specimen block at the bottom, a droplet of liquid was introduced closer to the surface with a syringe, and the surface was restored to its original smooth surface by the rinsing of the droplet. The dust is washed clean from DCA5-ZrO_2_, as shown in Figure 9, indicating the surface of DCA5-ZrO_2_ exhibits good self-cleaning performance.

### 2.3. Droplet Freezing Experiments

The droplet freezing process is a process from water droplets to ice, which needs to go through the following two stages: pre-cooling and droplet nucleation; the droplet nucleation will grow rapidly from bottom to top until the ice tip is formed. The droplet freezing test in this study was conducted at an air humidity of 26 ± 3%, an ambient temperature of 23 ± 3 °C, and a cold surface temperature of −10 °C. A set of droplets in the superhydrophobic coating was frozen at a temperature of −10 °C.

**Figure 10:**
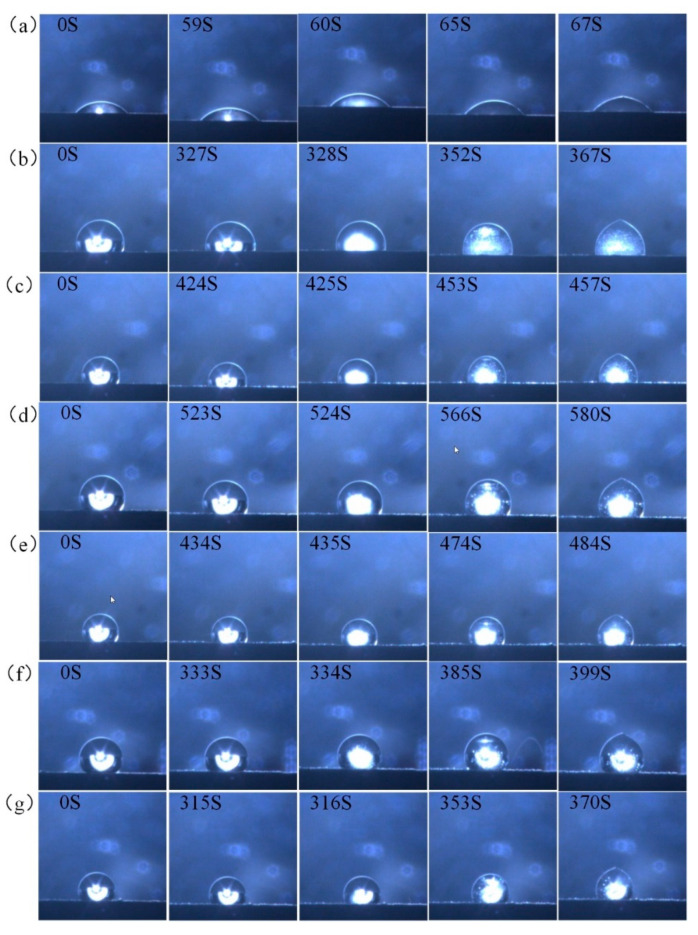
Droplet freezing test results on various surfaces. (**a**) TA2 surface freezing, (**b**) DCA3-ZrO_2_ coating surface freezing, (**c**) DCA4-ZrO_2_ coating surface freezing, (**d**) DCA5-ZrO_2_ coating surface freezing, (**e**) DCA6-ZrO_2_ coating surface freezing, (**f**) DCA7-ZrO_2_ coating surface freezing, and (**g**) DCA8-ZrO_2_ coating surface freezing.

A set of droplets freezing in the superhydrophobic coating is shown in Figure 10; Figure 10a is the TA2 pure titanium droplet freezing test, titanium has good thermal conductivity, the droplet freezing time took 67 S, and the droplet phase transition at 59 S releases the latent heat, which causes the change of the image. Figure 10b–g depict the droplet freezing times of DCA3–8-ZrO_2_, showing the morphological changes observed during the freezing of individual droplets. The prolongation of droplet freezing time on the superhydrophobic surface is due to the existence of an air layer between the surface and the droplets and the surface is converted from the Cassie–Baxter state to the Wenzel state with the prolongation of the freezing time, which is one of the reasons for the prolongation of droplet freezing time on superhydrophobic surfaces. This is an important factor for the superhydrophobic surface to prolong the droplet freezing time and a decrease in contact angle during the pre-cooling and freezing of the droplet can be observed in the figure. From the results in the experiment, the DCAx-ZrO_2_ surface is analyzed to have good ice suppression ability.

**Figure 11:**
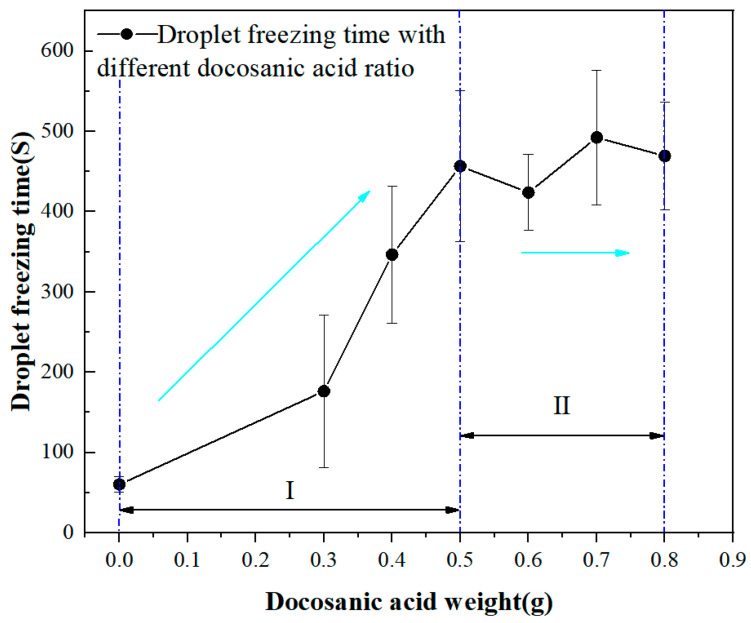
Freezing time of droplets on superhydrophobic surfaces with different ratios.

To ensure the accuracy of the test, each sample underwent three repeated droplet freezing tests and the average value of droplet freezing was recorded, as shown in Figure 11. The droplet freezing time on the TA2 surface at a cold surface temperature of –10 °C was 60.3 s. The droplet freezing time on the DCA3-ZrO_2_ hydrophobic surface was recorded as 173.3 s. The droplet freezing time on the DCA4-ZrO_2_ superhydrophobic surface was determined as 346.7 s. The droplet freezing time on the DCA5-ZrO_2_ surface was 456.7 s, while the droplet freezing time for DCA6-ZrO_2_ was 424 s. The time for droplet freezing in the case of the DCA7-ZrO_2_ surface was 469.3 s. For the DCA7-ZrO_2_ surface, the droplet freezing time was observed as 492.3 s. In the case of DCA8-ZrO_2_, 469.3 s was observed as the droplet freezing time.

All droplet freezing times on the DCAX-ZrO_2_ surfaces exceeded that for the TA2 surface. Upon analysis of the experimental data, it is evident that the longest droplet freezing time occurred on the DCA7-ZrO_2_, reaching 492.3 s. The variation in droplet freezing time between the DCA6-ZrO_2_ and DCA8-ZrO_2_ superhydrophobic surfaces compared to the DCA5-ZrO_2_ superhydrophobic surfaces is within 10%, exhibiting minor fluctuation changes. Additionally, the contact angle of DCA5-ZrO_2_ surpasses that of the superhydrophobic surfaces prepared with other ratios. DCA5-ZrO_2_ is a cut-off point for droplet freezing, in front of which the droplet freezing time tends to increase and after which the droplet freezing time remains stable. From the above data, it is inferred that DCA5-ZrO_2_ is the optimal ratio.

## 3. Experimental Section

### 3.1. Materials

White zirconium dioxide powder (particle size = 20 nm) was purchased from Shanghai Yaoge Alloy Materials Co. (Shanghai, China). DCA (purity = 95%) was purchased from Shanghai Bide Pharmaceutical Technology Co. (Shanghai, China). A pure TA2 titanium cube (30 mm × 30 mm × 3 mm) was purchased from Baoji Shengda Xing Metal Material Co. (Baoji, China). The development of anti-icing coatings is an important task. They are of great interest for use in aviation, aerospace, shipbuilding, and other industrial sectors. In particular, such coatings can improve the performance properties of parts. The pure commercial titanium widely used in industry is TA2, due to its corrosion resistance and moderate mechanical properties.

### 3.2. Preparation of the Superhydrophobic Surface

Superhydrophobic surface preparation is first sanded with 800 mesh sandpaper, smoothed, cleaned, and the surface is allowed to dry, followed by cleaning the spray gun and cleaning the surrounding environment. In the spraying, the epoxy resin primer is sprayed first, followed by the modified ZrO_2_ solution, before being dried in a drying oven at 80 °C after spraying. An air compressor (model Eluan750A) was purchased from Zhejiang Yongyuan Electromechanical Manufacturing Co., Ltd. (Taizhou, China). A paint spray gun on spout nozzle W-71 was used. An electric blast drying box was purchased from Shanghai Boxun Industrial Co. Medical Equipment Factory (Shanghai, China). Magnetic turntable equipment was purchased from Changzhou Jintan Liangyou Instrument Co. (Changzhou, China).

During the surface preparation process, 6 g of ZrO_2_ and ethanol were used to prepare a solution of approximately 40 mL and 0.1, 0.2, and 0.3 were added to increase the concentration of DCA to 0.8 g, respectively. Then, they were stirred in a magnetic turntable to allow for sufficient reaction and dissolution. Considering that DCA is slightly soluble in ethanol, an equilibrium state of saturated dissolution would occur when the modified substance solution is in a saturated state. Therefore, the modification time is 6–8 h. In order to ensure the uniform dispersion of ZrO_2_ particles during the stirring process, it is necessary to take out the stirred beaker from time to time to observe whether there is obvious deposition. If there is obvious deposition, the position should be adjusted in a timely manner to ensure that its micro and nanoparticles are evenly dispersed in the solution. After modification, the nanomaterial is named DCAx-ZrO_2_, where x represents the added DCA modified substance, such as 1 representing 0.1 g of DCA material, and so on.

During the coating preparation process, a two-step spraying method is adopted. Firstly, the primer is sprayed as the adhesive and, secondly, the modified zirconia solution is sprayed as the topcoat. The primer is prepared in a 1:1 ratio of epoxy resin adhesive and polyamide resin as curing agent in an ethanol solution, stirred for 30–45 min, and then evenly mixed before spraying. After spraying epoxy resin adhesive, it was placed in an electric hot air drying box at 60 °C for 3–5 min to reduce bubbles and ethanol in the primer, before spraying the topcoat. After the topcoat is sprayed, it was placed in an electric hot air drying box at 80 °C for 60 min to dry and to allow the ethanol to evaporate.

### 3.3. Characterization

Contact angle measurements were performed at room temperature using a contact angle meter (model SDC-350; Dongguan Shengding Precision Instrument Co. (Dongguan, China)). During the measurement, 4 μL of deionized water was dropped onto the sample and at least five points were measured on the same plane. The average value of these points was used for determining the contact angle and sliding angle of the superhydrophobic surface. The sliding angle was measured by dropping a droplet on the surface and using SDC-350 measures of the contact angle of the droplet when it is sliding on the surface as the sliding angle.

Scanning electron microscopy (SEM, EDS) was performed using an FEI-F50 scanning electron microscope. For SEM analysis, non-metallic materials were sprayed with gold. X-ray diffraction (XRD) was performed using Bruker D8 Advance. Fourier Transform infrared (FTIR) spectroscopy was performed using a NICOLET is 10. Thermogravimetric Analysis (TGA) was performed using STA449F3-QMS403D, manufactured by NETZSCH Instrument Manufacturing GmbH.

### 3.4. Analysis of Self-Cleaning Performance

The self-cleaning of the sample was performed using soil crushed into a powder. This crushed soil powder was scattered onto the superhydrophobic surface of the sample. The dimensions of the test sample were 30 mm × 30 mm × 3 mm. The test sample was tilted in the Petri dish. In the middle of the test sample, liquid droplets were dropped on the surface using a syringe, to observe the performance of dust on the sample surface.

### 3.5. Droplet Freezing Experiments

The droplet is placed on the test surface and the surface is cooled from room temperature to 0 °C, until it reaches −10 °C. The droplet is then held at −10 °C for droplet freezing tests, which are timed from 0 °C as droplet freezing test data, with the ambient temperature being room temperature and 5 μL of deionised water being used to freeze the droplet. Three tests were performed for each sample and the results were averaged. During the test, an industrial camera was used to capture photographs. A semiconductor refrigeration table was used for cooling. A thermally conductive silicone grease was coated on the top of the refrigeration table to facilitate rapid heat transfer.

## 4. Conclusions

The ratio between ZrO_2_ and DCA was studied and a superhydrophobic surface was prepared using a two-step spray method, with a contact angle of 154° and a sliding angle of 4°. The results of FTIR and TGA showed a good binding performance between DCA and ZrO_2_. The pyrolysis temperature of DCA5-ZrO_2_ was significantly increased compared to DCA and the proportion of C atoms in DCA5-ZrO_2_ was 65.22%. The self-cleaning ability of the surface was verified through self-cleaning tests and the surface had a good ice suppression ability through droplet freezing tests. The freezing time of droplets on the DCA5-ZrO_2_ surface is 6–8 times longer compared to the clean TA2 surface. Summary–Zirconia superhydrophobic surfaces have good self-cleaning and anti-icing effects, as well as having broad application prospects.

## Data Availability

Data are contained within the article.
